# Identification of urinary bacterial genes as biomarkers for non-invasive diagnosis of renal lupus

**DOI:** 10.1186/s40364-025-00828-5

**Published:** 2025-09-26

**Authors:** Virginia Pérez-Carrasco, Ana Soriano-Lerma, Cinzia Guzzi, María Luisa García-Martín, María J. Tello, Ángel Linde-Rodríguez, Victoria Sánchez-Martín, Matilde Ortiz-González, PRECISESADS Clinical Consortium, Lorenzo Beretta, Barbara Vigone, Jacques-Olivier Pers, Alain Saraux, Valérie Devauchelle-Pensec, Divi Cornec, Sandrine Jousse-Joulin, Bernard Lauwerys, Julie Ducreux, Anne-Lise Maudoux, Carlos Vasconcelos, Ana Tavares, Esmeralda Neves, Raquel Faria, Mariana Brandão, Ana Campar, António Marinho, Fátima Farinha, Isabel Almeida, Miguel Angel Gonzalez-Gay Mantecón, Ricardo Blanco Alonso, Alfonso Corrales Martínez, Ricard Cervera, Ignasi Rodríguez-Pintó, Gerard Espinosa, Rik Lories, Ellen De Langhe, Nicolas Hunzelmann, Doreen Belz, Torsten Witte, Niklas Baerlecken, Georg Stummvoll, Michael Zauner, Michaela Lehner, Eduardo Collantes, Rafaela Ortega-Castro, Mª Angeles Aguirre-Zamorano, Alejandro Escudero-Contreras, Mª Carmen Castro-Villegas, Yolanda Jiménez Gómez, Norberto Ortego, María Concepción Fernández Roldán, Enrique Raya, Inmaculada Jiménez Moleón, Enrique de Ramon, Isabel Díaz Quintero, Pier Luigi Meroni, Maria Gerosa, Tommaso Schioppo, Carolina Artusi, Carlo Chizzolini, Aleksandra Zuber, Donatienne Wynar, Laszló Kovács, Attila Balog, Magdolna Deák, Márta Bocskai, Sonja Dulic, Gabriella Kádár, Falk Hiepe, Velia Gerl, Silvia Thiel, Manuel Rodriguez Maresca, Antonio López-Berrio, Rocío Aguilar-Quesada, Héctor Navarro-Linares, Yiannis Ioannou, Chris Chamberlain, Jacqueline Marovac, Marta Alarcón Riquelme, Tania Gomes Anjos, José Gutiérrez-Fernández, Marta E. Alarcón-Riquelme, Miguel Soriano, Concepción Marañón, José A. García-Salcedo

**Affiliations:** 1 GENYO. Center for Genomics and Oncological Research Pfizer/University of Granada/Andalusian Regional Government, Avenida de La Ilustración 114, Granada, 18016 Spain; 2 Instituto de Investigación Biosanitaria Ibs.GRANADA, Granada, Spain; 3 Microbiology Unit, University Hospital Virgen de Las Nieves, Granada, 18014 Spain; 4 Department of Physiology (Faculty of Pharmacy, Campus Universitario de Cartuja), Institute of Nutrition and Food Technology “José Mataix”, University of Granada, Granada, 18071 Spain; 5 Biomedical Magnetic Resonance Laboratory-BMRL, Andalusian Public Foundation Progress and Health-FPS, Seville, 41092 Spain; 6 Instituto de Investigación Biomédica de Málaga y Plataforma en Nanomedicina-IBIMA Plataforma BIONAND, C/Severo Ochoa, 35, Malaga, 29590 Spain; 7 Center for Intensive Mediterranean Agrosystems and Agri-Food Biotechnology (CIAIMBITAL), University of Almeria, Almería, 04001 Spain

**Keywords:** Lupus, Renal lupus, Microbiome, Urobiome, Biomarker, Metagenomic

## Abstract

**Background:**

Systemic lupus erythematosus (SLE) is a complex autoimmune disease that often affects the kidneys, causing lupus nephritis. Diagnosis of this affection currently relies on kidney biopsy, an invasive and complex procedure. This study explores the diagnostic value of biomarkers based in the urobiome – the microbial community of the urinary tract – in patients with renal SLE.

**Methods:**

This study enrolled 585 female subjects including Healthy controls, non-renal and renal SLE patients. The taxonomic and functional differences of the urobiome in patients with SLE, as well as in the metabolites of interest, were identified by 16S rRNA profiling with PICRUSt functional inference and nuclear magnetic resonance (NMR). The accuracy of the identified biomarkers was tested by building random forest (RF) classification models. Furthermore, the results were validated in an independent cohort composed by 30 controls, 30 non-renal and 30 renal SLE patients.

**Results:**

Bacterial gene-based biomarkers with an AUC value of 0.7 ± 0.07 and 0.67 ± 0.07 to distinguish renal from non-renal SLE cases were identified. These biomarkers were validated in a validation cohort using quantitative PCR (qPCR), demonstrating their robust diagnostic performance. Furthermore, our analysis uncovered significant urobiome dysbiosis and distinct bacterial functional profile in both groups of SLE patients, with notable differences in amino acid metabolism pathways, particularly those involving valine and leucine, which were assessed by NMR-based urinary metabolite quantification.

**Conclusions:**

Some bacterial genes have been identified in the urobiome of SLE patients that allow differentiation between those with renal and non-renal lupus. These findings offer valuable insight into the association between the urobiome and SLE presentation, and lay the foundation for developing novel diagnostic tools that overcome the limitations of current methods, thereby improving patient care.

**Supplementary Information:**

The online version contains supplementary material available at 10.1186/s40364-025-00828-5.

## Background

Systemic lupus erythematosus (SLE) is a complex autoimmune disease characterized by inflammation and eventually dysfunction of multiple tissues and organs, such as skin, joints, brain or kidney. Hyperactivation of the immune response and aberrant auto-antibody production are characteristic of this pathology [1]. The global incidence of SLE is estimated to be around 5 per 100,000 person-year, with 0.40 million new cases diagnosed annually [2]. Although this pathology occurs in both genders, a higher prevalence has been observed in women of reproductive age [3]. One of the most severe clinical complications associated with SLE is nephritis, being also one of the main causes of death in these patients. Routine screening of SLE patients includes serum creatinine measurement, estimated glomerular filtration rate (eGFR) and urinalysis (proteinuria, haematuria and active urinary sediments) [4]. However, these assessments of kidney function are not specific to renal SLE, and may be common to other diseases that affect renal function. A kidney biopsy, an extremely invasive diagnostic method, is necessary to confirm the diagnosis of lupus nephritis [5]. Therefore, new biomarkers are needed for the early diagnosis and monitoring of renal involvement in SLE, with urinary biomarkers being particularly promising due to their non-invasive nature.

In recent years, metagenomics – e.g., with 16S ribosomal RNA (rRNA) gene sequencing—has become an essential tool for studying the microbiome of different body regions, as well as its relationship with different diseases. Microbiome analysis is very useful to identify biomarkers for the classification and prediction of different diseases, such as cancer [6, 7], neurodegenerative [8] or autoimmune diseases [9], among others. Gut and oral microbiota has been linked with the pathogenesis of several autoimmune diseases, including SLE [10–12]. Different studies have shown the presence of an intestinal dysbiosis in SLE patients, including those with lupus nephritis [13], as well as a SLE-associated alteration in intestinal barrier integrity [14–16]. The microbiota of other body areas, such as skin, has also been widely studied and the presence of a skin dysbiosis has also been identified in SLE patients [17, 18]. However, to date, only one study has approached the urobiome through the analysis of SLE-associated bladder microbial community [19], but the number of patients and the conclusions are very limited. Therefore, further studies are needed to investigate the value of urobiome-related biomarkers in this autoimmune disease.

In this study, we aimed to characterize the urinary bacterial community in a large cohort of 585 individuals – including healthy controls, non-renal and renal SLE patients – to identify microbial biomarkers with potential diagnostic value for renal involvement in SLE.

## Methods

### Patient recruitment and sample collection

Mid-stream urine samples were obtained from the European PRECISESADS project, a multi-centre study with 9 countries involved. Patients included in this study were diagnosed according to international established criteria for SLE [20]. Based on clinical data, recruited SLE patients were classified into two subgroups: (1) non-renal lupus patients: subjects with a diagnosis of lupus but without kidney involvement; and (2) renal lupus patients: subjects with a diagnosis of lupus and renal involvement, defined as the presence of abnormal creatinine levels (serum creatinine levels ≥ 20% ULN, increase in serum creatinine levels ≥ 50% *vs* baseline or reduced GFR < 60 ml/min), abnormal urine protein/creatinine ratio (≥ 0.2) and/or proteinuria (≥ 500 mg/24 h). Individuals with menstruation, active infection or antibiotic treatment were excluded. Clinical, serological and demographic data were gathered from all included subjects. All participants provided a written informed consent, previously approved by a local ethics committee for each centre, for inclusion in the study.

We included 339 healthy controls and 336 SLE patients (236 non-renal and 100 renal SLE patients). These samples were divided into a discovery cohort (309 healthy controls, 206 non-renal and 70 renal SLE patients) and a validation cohort (30 healthy controls, 30 non-renal and 30 renal SLE patients) (Supplementary Table 1). Urine samples were centrifuged, and supernatants and bacterial pellets were stored at −80ºC until further processing.

### Bacterial DNA extraction, 16S library preparation and high-throughput sequencing

DNA isolation was performed as previously described [21], with some modifications. Briefly, urine pellets were dissolved in 100 µL of lysis buffer (10% w/v sodium dodecyl sulphate in H2O, 0.5 M EDTA, pH 8.0, 5 M sodium chloride) and 0.4 M guanidine thiocyanate at 68ºC for one hour. Next, 2 M ammonium acetate were added and the protocol outlined in [20] was followed from this step onward. DNA integrity and quality were evaluated and quantified using a spectrophotometer (NanoDrop 2000 UV–Vis; Thermo Fisher Scientific Inc.). Negative controls were included in all extraction batches to ensure the absence of contamination. Isolated DNAs were stored at −20ºC until library preparation.

PCR amplification products of the V1-V3 hypervariable regions of the 16S rRNA gene were obtained using fusion universal primers 27 F (Illumina adaptors + 5’AGAGTTTGATCMTGGCTCAG3’) and 533R (Illumina adaptors + 5’TTACCGCGGCKGCTGGCACG3’). Similarly, negative controls were included in each PCR batch to ensure the absence of contamination. Amplicon multiplexing and sequencing was carried out using a dual indexing tag-tailed design with 8nt indices from Nextera XT Index Kit v2 (Illumina, San Diego, CA, USA). Paired-end sequencing of the 16S rRNA amplicon libraries was conducted on the Illumina MiSeq platform using v3 kit chemistry (300 + 300 bp). The raw sequencing data are available at the Sequence Read Archive (SRA) of the National Centre of Biotechnology Information (NCBI) under the Bioproject accession number PRJNA1237824.

### Data preprocessing

Bioinformatics analysis and quality filtering were carried out using Mothur software v1.39.5 (University of Michigan Medical School, Ann Arbor, MI, USA) [22]. Chimeric reads were identified and excluded using Chimera UCHIME. Diversity was assessed through operational taxonomic units (OTUs) at 3% dissimilarity, employing the distance-based greedy clustering algorithm (dgc), calculating the coverage, number of observed OTUs and richness index Chao1 after rarefaction. Redundant, non-chimera FASTA files were taxonomically classified using SILVA v132 database. Abundance was expressed as a percentage relative to the total number of sequences in each sample. For statistical analysis, only bacterial taxa with a total abundance higher than 0.01% and present in at least 20% samples were considered. Functional analysis was performed by Phylogenetic Investigation of Communities by Reconstruction of Unobserved States (PICRUSt) analysis, based on high-throughput 16S rRNA gene sequencing data [23]. Gene families were then regrouped according to the Kyoto Encyclopedia of Genes and Genomes (KEGG) orthologues database.

### Confounder analysis

To identify potential confounding variables, we first compared clinical features between study groups using the Mann–Whitney U-test for continuous variables and Fisher’s exact test for categorical variables. Significantly different variables between groups (*p* < 0.05) were considered potential cofounders and included in the subsequent multivariate analysis. To assess the proportion of microbiome compositional variance explained by the study group factor and these selected confounding factors, we performed a PERMANOVA test based on Bray–Curtis distances and implemented in R using adonis() function form the vegan R package. Variance explained (R^2^) were extracted for each variable and evaluated.

### Statistical analysis

After checking for the absence of normality in the diversity indices and Firmicutes/Bacteroidetes ratio with the Shapiro–Wilk test, the non-parametric Mann–Whitney U-test was applied using GraphPad Prism version 9.0.0 (GraphPad Software, San Diego, California, USA). The similarity of the bacterial communities between samples was evaluated by a multivariant PERMANOVA test based on Bray–Curtis distances. This statistical test was implemented in PRIMERe Permanova + (PRIMER-E Ltd, Plymouth, UK) with square root transformation and permutation of residuals under a reduced model (9999 permutations). For all statistical analyses, a significant threshold of *p* < 0.05 was applied.

Selection of differentially abundant OTUs or genes with higher importance in the variance between the study groups was carried out by Linear discriminant analysis Effect Size (LefSe) via Python 3.12.4, considering a *p* < 0.05 and a linear discriminant analysis (LDA) value of 2.0 as significant [24]. The specific bacterial species corresponding to each differential OTU was identified through a BLAST analysis.

Microbiome Associations with Lineal Models (MaAsLin2) [25] was employed to identify significant altered bacterial pathways in SLE groups, analysis implemented in R. Statistically significant KEGG orthologues (KO) were identified using *edgeR* package and *p*-adjusted values < 0.05. Selected KOs were mapped to KEGG pathways and pathways of interest were coloured according to log_2_ fold change (log_2_FC) values using KEGG Mapper Color online tool.

Spearman correlations between identified biomarkers and clinical parameters were implemented in R using the *rcorr* function.

### Classification model construction and feature importance

Random forest (RF) classification models were constructed to distinguish non-renal and renal SLE patients from healthy controls. The models were developed using the scikit-learn (v1.5.0) package with stratified tenfold cross-validation to configure training and testing data sets. Each model consisted of 1000 estimator trees, with each tree utilizing 10% of the total features. To evaluate the impact of individual features on predictions, we assessed the importance of each included feature identifying the top-ranking biomarkers of each model. Finally, the performance of the models was evaluated using the AUC-ROC, accuracy and precision metrics.

Differential bacterial genes belonging to the bacterial metabolic pathways of interest were also evaluated for their potential as biomarkers. Using a similar methodology, the AUC, accuracy and precision of each of these genes was calculated and those with a higher AUC value were selected as biomarkers.

### qPCR validation

To quantify the abundance of the orthologous genes from the selected metabolic pathway, qPCR analysis was performed on 30 healthy controls, 30 non-renal SLE and 30 renal SLE samples. Initially, the contribution of each OTU to each selected gene was assessed. This contribution was defined as the ratio of the contribution of each OTU for a given gene to the total contribution of all OTUs in this gene. OTUs that contributed the most to each gene and exhibited differential abundance between the study groups were selected. To identify the specific bacterial species associated with the selected OTUs, a BLAST analysis was conducted. Specific primers were designed for the most contributing bacterial species corresponding to each selected gene. The primers listed in Supplementary Table 2 were used for candidate genes while standard primers F908 and R1096 were employed for 16S rRNA. qPCR reactions were prepared using a final primer concentration of 0.5 µM, 5 ng of gDNA and SYBR Green qPCR mix (4,309,155, Thermo Fisher Scientific, USA) in a total volume of 20 µl. Cycling conditions consisted of an initial pre-denaturation at 95ºC for 10 min and 40 cycles consisting of denaturalization at 95ºC for 15 s and annealing-extension at 60ºC for 1 min. Gene abundance was calculated using 2^−ΔΔCt^ method, comparing Ct values of candidate genes to 16S rRNA Ct values. Statistical analysis was performed using a two-sided Wilcoxon rank-sum test.

Furthermore, the abundance of these genes, along with those genes included in the general classification model, was quantified in the validation cohort following the procedure described above.

### NMR-based urinary metabolite quantification

Urinary supernatants from a subset of 400 subjects were thawed at room temperature for 20–30 min, centrifuged, and mixed with a buffer solution containing phosphate buffer (pH = 7.4), TSP (3-(trimethylsilyl-)3,3,2,2-tetradeutero-propionic acid), and D2O (final concentration 10%). 1H NMR spectra were acquired at 300 K using a Bruker Avance 600 MHz spectrometer equipped with a nitrogen cooled cryoprobe (Prodigy TCI). The 1D Nuclear Overhauser Effect SpectroscopY (NOESY) presat sequence was applied. Additionally, 2D J-resolved spectra were acquired to assist in metabolite identification. The acquisition was carried out under standard operating procedures with automatic adjustments for temperature, tuning, shimming, and radiofrequency pulse for each sample. Exponential filtering (0.3 Hz), Fourier transformation, phase, and baseline correction were performed using TopSpin 3.5.pl7 (Bruker BioSpin, Ettlingen, Germany). The Electronic Reference to access In-vivo Concentrations (ERETIC), as implemented in TopSpin 3.5.pl7 (ERETIC 2), was used as a concentration reference. Metabolite identification and quantification of urine samples (controls and SLE patients) were performed with Chenomx Profiler software (v10.0, Chenomx Inc., Edmonton, Ca) using the Chenomx 600 MHz (v11) database and the human metabolome database (HMDB). To determine differences between controls and patient groups (non-renal and renal SLE), the concentration of the selected metabolites was normalized to formate concentration and statistically analyzed using the non-parametric Mann–Whitney test.

## Results

### Demographic and clinical characteristics of study participants and cofounder identification

The study comprised 585 mid-stream urine samples from female subjects, 309 healthy controls and 276 samples from SLE patients (206 non-renal SLE subjects and 70 renal SLE subjects). Additionally, a validation cohort was included, consisting in 30 healthy controls, 30 non-renal and 30 renal SLE patients, all women. All subjects in the study groups had similar ages. Demographics and clinical characteristics of study participants are included in Supplementary Table 1.

16S amplicon libraries of all samples were sequenced using the MiSeq platform (Illumina) obtaining a total number of 11,059,442 reads after elimination of misaligned sequences and chimeras. The sequencing depth (an average of 18,900 sequencing reads in each sample), was sufficient to obtain coverages greater than 99%, so they were used for further analysis.

Statistical analysis of clinical variables showed significant differences between study groups in disease duration (*p* = 0.002), immunosuppressant use (*p* = 0.0001) and heart involvement (*p* = 0.0001) (Supplementary Table 1). These variables were evaluated as potential cofounders. However, the variance explained (R^2^) by these factors was much lower than that obtained for the study group factor (Supplementary Figure 1). Additionally, the variance of the samples based on these clinical factors was not shown to be significant. These results revealed that the study group factor showed a predominant value on the variance of the samples without having to take these clinical cofactors into account.

### Altered urobiome diversity and taxonomic composition in SLE patients

**Fig. 1:**
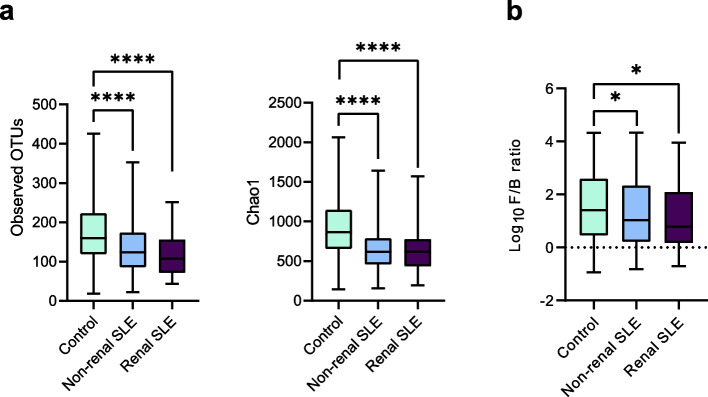
Alterations of urobiome diversity in SLE. **a** Alpha-diversity indices (observed species and Chao1) in the study groups (healthy controls, non-renal SLE and renal SLE patients). **b** Firmicutes/Bacteroidetes ratio of the urobiome in the study groups. Differences between groups were assessed by Mann–Whitney U-test (* *p* < 0.05; **** *p* < 0.0001)

We first assessed alpha diversity in SLE patients and healthy controls, differentiating between non-renal and renal SLE groups. A significant reduction in diversity, evaluated by the number of observed OTUs (*p* < 0.0001) and the Chao1 index (*p* < 0.0001), was observed in both non-renal and renal SLE patients (Fig. 1a).

**Fig. 2:**
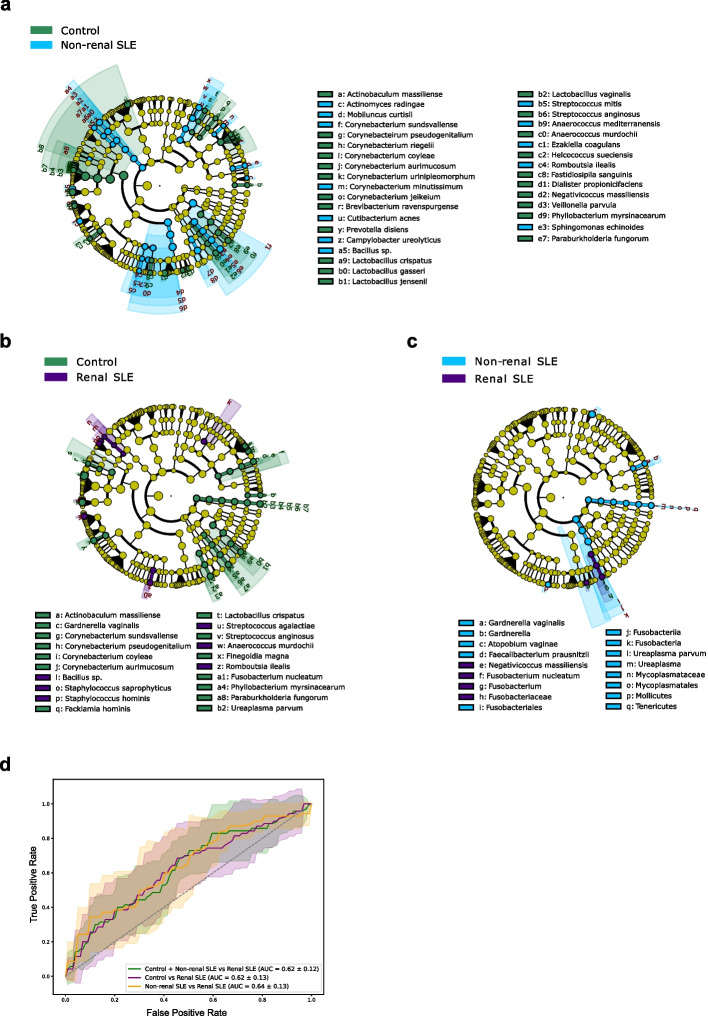
Urinary bacterial species discriminating non-renal SLE and renal SLE. **a**-**b**-**c** Linear discriminant analysis Effect size (LEfSe) of non-renal (**a**) and renal (**b**) SLE patients compared to healthy controls, and between both groups of patients (**c**). Cladogram shows differentially distributed bacterial taxa (*p* < 0.05, LDA > 2.0). Taxonomic features are represented in a hierarchical structure, with higher taxonomic levels oriented towards the inner part of the plot. Taxa with significant differences are coloured according to whether they are enriched in controls (green), non-renal SLE (blue) or renal SLE (purple); yellowish green for non-significant. **d** ROC curves of the optimized RF model constructed with a set of 14 bacterial species to differentiate renal SLE patients from non-renal SLE and controls. Mean AUC and standard deviation of stratified tenfold cross-validation are shown

The bacterial composition of the urobiome in SLE patients was also altered. At phylum level, the Firmicutes/Bacteroidetes ratio showed a significant variation (*p* < 0.05), with a decrease in Firmicutes abundance and an increase in Bacteroidetes in all SLE patients (Fig. 1b). Furthermore, PERMANOVA analysis revealed differences (*p* = 0.0035) in the urobiome composition at OTU level between the three groups, evidencing variations in microbial profiles. To identify bacterial taxa differentially enriched among the groups, Linear discriminant analysis Effect Size (LefSe) was performed. This analysis identified 34 OTUs that were differentially enriched and displayed an LDA value > 2 between healthy controls and non-renal SLE patients (Fig. 2a). Specifically, 22 OTUs were reduced in these patients, including several *Corynebacterium* species (*C. sundsvallense, C. pseudogenitalium, C. riegelii, C. aurimucosum, C. urinipleomorphum* and *C. jeikeium*), several *Lactobacillus* species (*L. crispatus, L. gasseri, L. jensenii* and *L. vaginalis*), as well as *Streptococcus anginosus* and *Veillonella parvula*, among others. Conversely, 12 OTUs were enriched in non-renal SLE patients and they were identified as *Cutibacterium acnes, Campylobacter ureolyticus, Streptococcus mitis, Romboutsia ilealis*, among others. In renal SLE patients, 20 OTUs showed differences in abundance compared to controls (Fig. 2b). Among them, 14 OTUs were reduced, including *Gardnerella vaginalis*, several *Corynebacterium* species (similar to those observed in non-renal SLE patients), *Lactobacillus crispatus, Streptococcus anginosus, Finegoldia magna, Fusobacterium nucleatum* and *Ureaplasma parvum*, among others. In contrast, 6 OTUs were increased in renal SLE and were assigned to *Bacillus sp., Staphylococcus saprophyticus, S. hominis, Streptococcus agalactieae, Anaerococcus murdochii, Romboutsia ilealis.* Additionaly*,* only 6 OTUs showed differences in abundance between non-renal and renal SLE patients. *Gardnerella vaginalis, Atopobium vaginae, Faecalibacterium prausnitzii* and *Ureaplasma parvum* were enriched in non-renal SLE, while N*egativicoccus massiliensis* and *Fusobacterium nucleatum* were enriched in renal SLE patients (Fig. 2c).

Among the differentially enriched OTUs, 22 were exclusive to non-renal SLE patients (Supplementary Fig. 2), including *Corynebacterium jeikeium, Campylobacter ureolyticus, Prevotella disiens* and *Dialister propionicifaciens*. In contrast, 5 OTUs were unique to renal SLE, such as *Streptococcus agalactiae* and *Finegoldia magna*. Additionally, *Atopobium vaginae* and *Faecalibacterium prausnitzii* were exclusively found in the differential non-renal versus renal SLE comparison. Interestingly, only 3 OTUs, *Ureaplasma parvum*, *Fusobacterium nucleatum* and *Gardnerella vaginalis*, were shared between the renal SLE versus controls and renal versus non-renal SLE.

### Bacterial taxa-based classification models for renal SLE

Once the differentially enriched OTUs between the study groups were identified, we constructed an optimized stratified tenfold cross-validation Random Forest (RF) model to distinguish renal from non-renal SLE and healthy controls. The model incorporated all unique and shared differential OTUs identified in the comparison between controls versus renal SLE and non-renal versus renal SLE. This model was optimized by selecting the bacterial species that provided the best classification performance for renal SLE patients. The highest Area Under the Curve of the Receiver Operating Characteristics curve (AUC-ROC) score was obtained including 8 unique and shared differential OTUs in the above-mentioned comparisons*.* Among these*, Finegoldia magna, Fusobacterium nucleatum, Ureaplasma parvum* and *Gardnerella vaginalis* were the top-ranking biomarkers in the model (Supplementary Fig. 3).

Thus, this optimized model demonstrated a moderate classification performance, with an AUC-ROC score of 0.62 (accuracy: 0.86, precision: 0.12), to identify renal SLE patients compared to the other study groups (Fig. 2d). When we used this model to differentiate renal patients from non-renal patients and healthy controls independently, AUC-ROC values of 0.64 (accuracy: 0.74, precision: 0.38) and 0.62 (accuracy: 0.80, precision: 0.28), respectively, were obtained. These results indicate the potential utility of the model in distinguishing renal involvement in SLE.

### A general gene-based classification model to distinguish renal SLE patients from non-renal SLE and healthy individuals

**Fig. 3:**
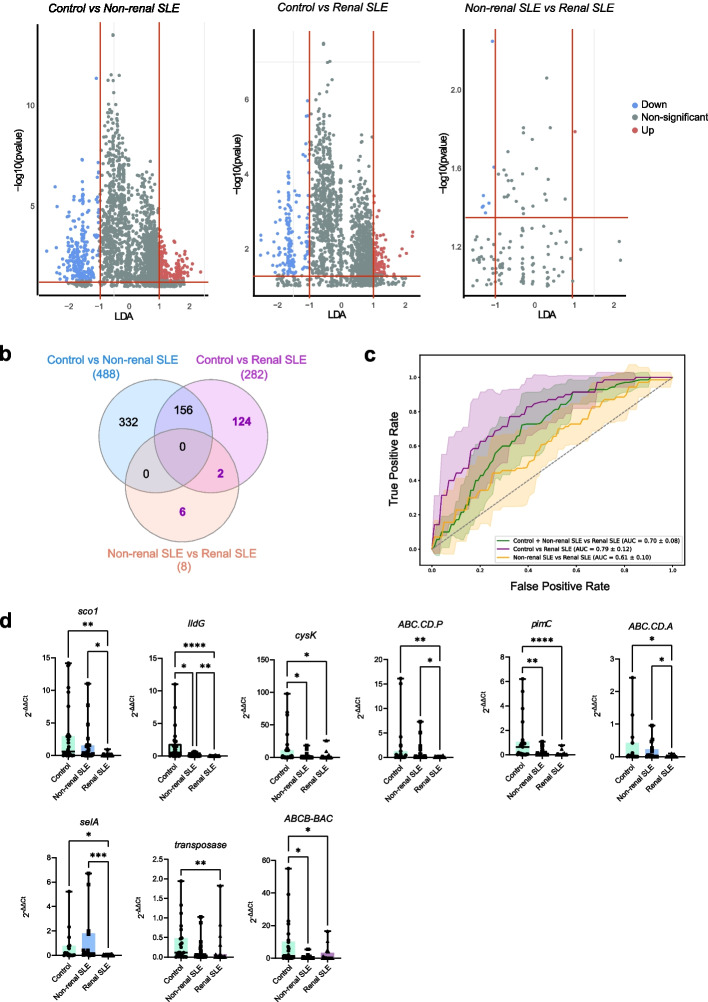
Construction of a general gene-based RF classification model to differentiate renal SLE patients from non-renal SLE and control subjects. **a** Linear discriminant analysis Effect size (LEfSe) of control vs non- renal SLE, control vs renal SLE and non-renal SLE versus renal SLE at KEGG orthologous genes level. Each gene is represented by a dot. Genes are colored based on whether they are significantly enriched (red, *p* < 0.05 and LDA > 1.0), significantly reduced (blue, *p* < 0.05 and LDA < −1.0), or not significantly altered (*p* > 0.05 and/or −1.0 > LDA < 1.0). **b** Venn diagram of the significant differentially enriched genes in the three comparisons. **c** Diagnostic performance of the optimized RF classification model based on selected differentially enriched genes. Graph represents mean AUC using this model to differentiate patients with renal lupus from each of the other groups. **d** Validation of the abundance of selected genes by qPCR in the validation cohort. *P* values were computed using Mann–Whitney U test (* *p* < 0.05, ** *p* < 0.01)

Bacterial functional profile of controls and SLE patients was estimated using Phylogenetic Investigation of Communities by Reconstruction of Unobserved States (PICRUSt). The KEGG (Kyoto Encyclopaedia of Genes and Genomes) ortholog (KO) composition of the samples was subjected to LEfSe analysis to identify differentially enriched genes in the study groups. Considering an LDA score > 1.0 and a *p-*value < 0.05, the analysis revealed 488 differentially enriched genes between non-renal SLE patients and controls, 282 differentially enriched genes between renal SLE patients and controls, and 8 altered between the two groups of lupus patients (Fig. 3a, Supplementary Table 3–5). To further refine the classification of SLE patients with renal involvement, we developed a bacterial gene-based stratified tenfold cross-validation RF model to distinguish renal SLE from non-renal SLE and healthy controls. We then selected unique and shared differential genes for renal SLE patients versus controls and non-renal versus renal SLE comparisons (Fig. 3b). Next, we built a stratified tenfold cross-validation RF model using the selected genes and identified the subset of genes that offers the best predictive performance for the model. The best combination of genes was selected through an iterative process that evaluates all possible feature combinations using AUC values. The final model included 9 bacterial genes (*sco1, lldG, cysK, ABC.CD.P, pimC, ABCB-BAC, selA, transposase, ABC.CD.A*) and achieved an AUC score of 0.7 (accuracy: 0.87, precision: 0.78) for distinguishing renal SLE patients from non-renal SLE patients and heathy controls (Fig. 3c). Notably, this model allowed a powerful classification of renal patients from controls too (AUC: 0.79; accuracy: 0.82, precision: 0.8) although its power was lower when differentiating between the two groups of SLE patients (AUC: 0.61; accuracy: 0.71, precision: 0.57). To confirm these results, we analysed an independent cohort comprising 30 controls, 30 non-renal and 30 renal SLE patients. We quantified the abundance of these nine genes by qPCR, demonstrating differential abundance in renal SLE patients compared to both non-renal SLE and healthy controls (Fig. 3d).

### Altered bacterial functions in SLE patients

**Fig. 4:**
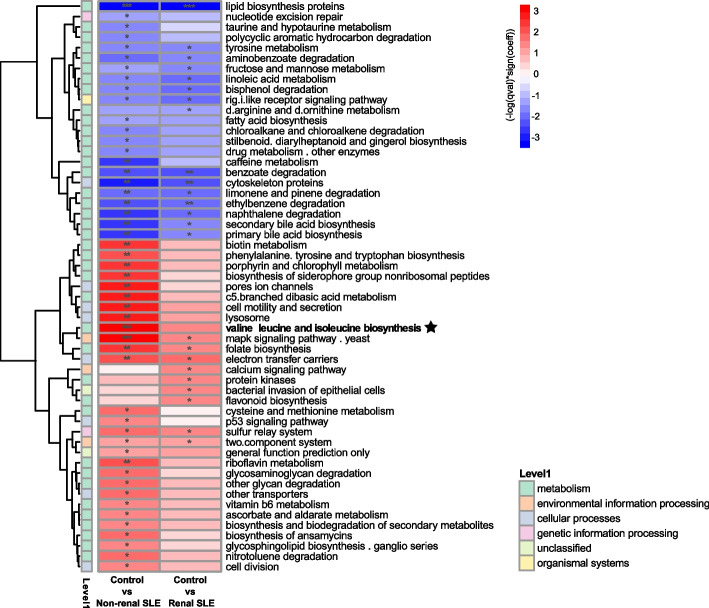
Functional alterations in non-renal and renal SLE patients. Heatmap of functional bacterial pathways differentially abundant in non-renal and renal SLE patients relative to controls, identified by Maaslin2 analysis. Significant pathways (*p* < 0.05) are marked with stars (*p < 0.05; **p < 0.01; ***p < 0.001). Association coefficient (- log(qval)*sign(coeff)) is indicated by color gradient. The association coefficient > 0: enriched in latter; association coefficient < 0: reduced in latter

Maaslin2 analysis was performed to identify differences in the abundance of KEGG bacterial pathways associated with non-renal and renal SLE patients relative to healthy controls. 55 pathways were identified with significant differences in abundance associated with non-renal and/or renal SLE patients. Among them, 23 pathways were decreased and 32 pathways were increased in SLE patients (Fig. 4). The number of pathways with significant differences in abundance relative to controls was higher in non-renal SLE patients than in renal SLE patients. SLE individuals without renal manifestations showed an enrichment of amino acid metabolism pathways (e.g., valine, leucine and isoleucine biosynthesis) and cofactors and vitamin metabolism (e.g., biotin metabolism). However, many of these pathways were not significantly altered in patients with renal damage.

**Fig. 5:**
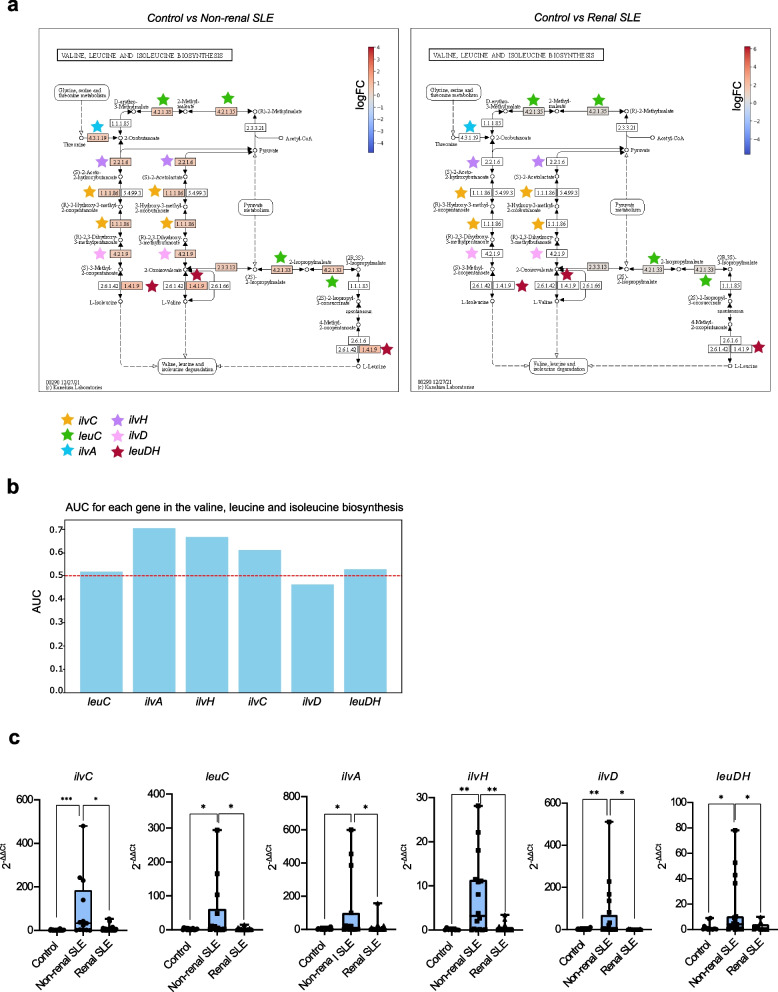
Alterations of the valine and leucine metabolism in non-renal and renal SLE patients. **a** Valine, leucine and isoleucine biosynthesis pathway map represented using KEGG Mapper Color tool. Differential gene abundance between non-renal or renal SLE and healthy controls was assessed by calculating logFC. Significant differences in genes abundance (*p*-adjusted < 0.05) between SLE groups and controls are coloured in the map with a color gradient and marked with stars. **b** Mean AUC value of the differentially enriched genes of this pathway and candidates as biomarkers of renal SLE versus non-renal SLE. **c** qPCR quantification of candidate genes in control (*n* = 30), non-renal SLE (*n* = 30) and renal SLE (*n* = 30) groups. *P* values were computed using Mann–Whitney U test (* *p* < 0.05, ** *p* < 0.01, *** *p* < 0.001)

**Fig. 6:**
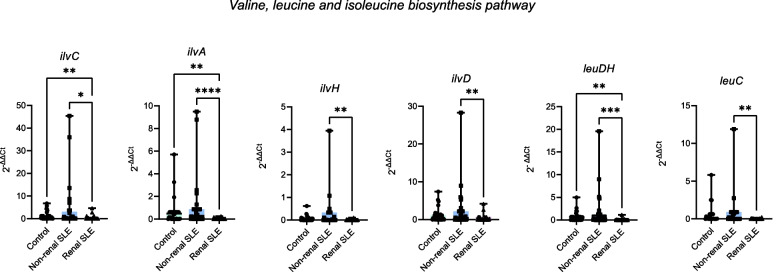
Validation of the abundance of the valine, leucine and isoleucine biosynthesis pathway genes in a new cohort. qPCR quantification of selected genes in control (*n* = 30), non-renal SLE (*n* = 30) and renal SLE (*n* = 30) groups. *P* values were computed using Mann–Whitney U test (* *p* < 0.05, ** *p* < 0.01)

We highlight the biosynthesis pathway for valine, leucine and isoleucine that was meaningfully altered in non-renal SLE but not in renal SLE individuals. These amino acids are involved in the regulation of the immune response and, therefore, in the development of autoimmunity [26]. Notably, the abundance of genes coding the main enzymes involved in this pathway were enriched relative to control in non-renal SLE patients, but not in SLE patients with renal damage (Fig. 5a). In fact, some of these genes, specifically *ilvA* and *ilvH*, were selected as the best biomarkers to distinguish renal from non-renal SLE by RF (AUC-ROC scores of 0.7 and 0.66, respectively) (Fig. 5b and Supplementary Fig. 4). However, the combination of both genes does not improve the ability to differentiate between the two groups of SLE patients (Supplementary Fig. 5). To confirm these findings obtained from PICRUSt data, we quantified the abundance of these bacterial genes in an aleatory subgroup of the discovery cohort by qPCR corroborating the results obtained by PICRUSt analysis. All key genes of this pathway were significantly enriched in non-renal SLE patients compared to those with renal damage as estimated by qPCR (Fig. 5c). To confirm the diagnostic potential of identified biomarkers, the selected genes of the valine, leucine and isoleucine biosynthesis pathway were validated in the independent cohort. Similar to the results obtained in the main cohort by PICRUSt and qPCR, these genes showed a higher abundance in non-renal SLE patients and could be considered as biomarkers to differentiate them from renal SLE individuals, further reinforcing their value as lupus biomarkers distinguishing between renal and non-renal forms of the disease (Fig. 6). Additionally, *ilvH* or *ilvA* abundance showed a significant inverse correlation with several circulating autoantibodies such as anti-dsDNA and anti-U1-RNP or anti-SSA-60, respectively (Supplementary Figure 6).

**Fig. 7:**
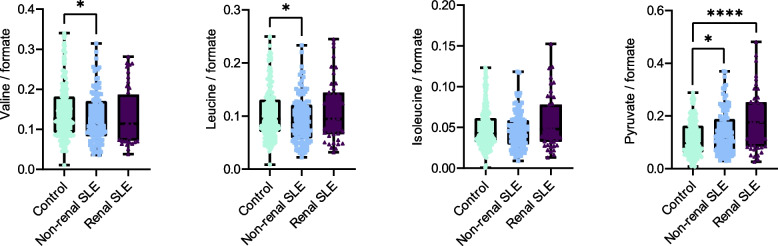
Alteration in the urinary valine, leucine and pyruvate concentrations in non-renal SLE patients. Initial and final urinary metabolites of the valine, leucine and isoleucine biosynthesis pathway were quantified by NMR. Urinary valine, leucine, isoleucine and pyruvate were normalized using urinary formate concentration. *P* values were computed using Mann–Whitney U test (* *p* < 0.05, ** *p* < 0.01, *** *p* < 0.001, **** *p* < 0.0001)

Finally, we quantified the urinary concentrations of these three amino acids in the study groups using NMR-based Metabolomics (Fig. 7). Interestingly, a significant reduction in valine and leucine urinary levels were observed in non-renal SLE patients in comparison with controls, while no significant changes were observed in SLE patients with renal involvement. In addition, urinary level of pyruvate, the initial metabolite of the pathway, was also significantly elevated in patients without renal manifestations. These results, together with the observed increase in the abundance of genes encoding enzymes of this pathway, suggest enhanced microbial-mediated degradation of valine and leucine into pyruvate, leading to reduced amino acid availability in this group of patients.

Similarly, the phenylalanine, tyrosine and tryptophan biosynthesis pathway was also altered in non-renal and renal SLE patients relative to controls (Fig. 4). The central part of this pathway is the shikimate pathway, which leads to the synthesis of tryptophan and indole, compounds also related with the regulation of the immune response [16]. As in the previous case, the genes coding for the main enzymes of this pathway were differentially enriched in patients with non-renal SLE but not in renal SLE (Supplementary Fig. 7a). In this case, *trpE* and *trpG* were key biomarkers of this pathway for the differentiation between the two groups of patients with an AUC score of 0.71 and 0.7, respectively (Supplementary Fig. 7b-c). These results collectively highlight the differential metabolic and genetic alterations of the urobiome associated with non-renal and renal SLE, particularly in pathways linked to immune regulation.

## Discussion

This study investigated taxonomic and functional bacterial alterations in the urobiome of SLE patients, focusing on differences between non-renal SLE subjects and those with renal manifestations of the disease. Importantly, it underscores the potential of bacterial biomarkers as non-invasive diagnostic tools, particularly for renal SLE.

Our work identified urinary bacterial genes, such as *ilvA* or *ilvH*, as potential biomarkers for renal lupus. They were identified in a large cohort of patients and subsequently confirmed in an independent cohort.

While other urinary biomarkers, such as MCP-1, VCAM-1, NGAL, ALCAM, among many others that have been shown promise for renal SLE, further studies are needed to confirm their specificity and accuracy in disease detection [27–29]. Additionally, certain gut bacterial species, such as *Enterococcus gallinarum* [16] or *Bacteroides acidifaciens* [30], have been implicated in the progression of SLE in mouse models. However, their utility as biomarkers has not been evaluated in patients. Moreover, no microbiota-based biomarkers specific to lupus nephritis have been identified to date.

Our study also revealed reduced diversity and a dysbiosis in the urobiome of SLE patients, with notable differences between non-renal and renal SLE. An alteration in a large number of bacterial species, as well as a reduction in the Firmicutes/Bacteroides ratio was observed in SLE patients compared to healthy controls (Figs. 1, 2). Firmicutes/Bacteroidetes (F/B) ratio is commonly used in microbiome studies. It refers to the relative abundance of the two mayor bacterial phyla in the human microbiome, so changes in this ratio can reflect a shift in the microbiome composition and functionality. These findings contrast with a previous study of the SLE bladder microbiome, which reported increased bacterial diversity in SLE patients and no difference between patients with and without lupus nephritis [19]. The discrepancy in results may be due to variations in the study design, such as differences in urine samples types, number and background of participants and other methodological factors. Of note, other studies analysing the gut microbiome of SLE patients have similarly observed reduced bacterial diversity and a decreased Firmicutes/Bacteroidetes ratio [31, 32].

In addition, we identified a set of altered bacterial species in non-renal SLE, as well as another set of differential species in renal SLE (Fig. 2a-c). Among the species uniquely altered in renal SLE patients, *Finegoldia magna*, reduced in renal SLE, was identified as the most important contributor to urobiome variation (Fig. Supplementary 3). Other key features included *Fusobacterium nucleatum* (enriched in renal compared to non-renal SLE) and *Ureaplasma parvum* and *Gardnerella vaginalis*, both reduced in renal SLE. Although *Finegoldia magna* is part of the urinary commensal microbiota, it has also been linked to enhanced inflammatory response [33]. Similarly, *U. parvum* and *G. vaginalis,* while typically commensal, can influence development of infections [34] and pro- and anti-inflammatory responses depending on the environment [35, 36]. Therefore, patients with renal lupus exhibit urinary dysbiosis characterized by reduced bacterial diversity and functional loss. On the other hand, *Fusobacterium nucleatum* has been previously identified as promoting the pro-inflammatory response in other diseases such as colorectal cancer [37], inflammatory bowel disease [38] or periodontal disease [39]. This bacterium has been linked to modulation of IL-17 expression and Th17 cell proliferation in the intestinal environment [40]. Therefore, some pro-inflammatory bacteria such as *F. nucleatum* might contribute to persistent inflammation in renal SLE patients, despite their observed reduced microbial diversity.

Bacterial genes are often considered better biomarkers than bacterial species due to the concept of functional redundancy in microbial communities. This phenomenon refers to the ability of multiple bacterial species to perform the same metabolic functions despite their taxonomic differences [41, 42]. Similar to our analysis at the species level, we identified bacterial genes that were differentially abundant in non-renal and renal SLE (Fig. 3a). Genes altered exclusively in renal SLE patients were used to construct a RF classification model including 9 genes, which outperformed the species-based model achieving an AUC of 0.7 (Fig. 3c). Notably, this model was particularly powerful in differentiating renal SLE patients from controls, though it was less effective at distinguishing between non-renal and renal SLE individuals.

A bacterial pathway analysis based on the taxonomic data allowed us to identify a significant increase in bacterial pathways related to amino acid metabolism in non-renal SLE patients, which were not altered in patients with renal affection (Fig. 4). Specifically, genes involved in the valine and leucine metabolism were significantly enriched in non-renal SLE patients (Fig. 5a). These branched-chain amino acids (BCAAs) have been associated with an enhanced autoimmune response [43] as they are critical for T cell activation and function. Leucine, for instance, can activate mTOR signalling, a pathway implicated in abnormal T cell activation in SLE [26]. Alterations in these BCAAs metabolism could thus influence T cell responses involved in lupus-associated autoimmunity. The increased expression of genes involved in valine and leucine metabolism in non-renal SLE patients was validated by direct quantification using qPCR (Fig. 5c), and confirmed in an independent cohort (Fig. 6). Furthermore, the genes of this pathway *ilvA* and *ilvH*, proved to be of strong diagnostic value for differentiating renal and non-renal SLE, with an average of AUC 0.7 and 0.67, respectively (Fig. 5b). Similarly, genes like trpE and *trpG*, involved in phenylalanine, tyrosine and tryptophan biosynthesis, also showed strong diagnostic potential for renal SLE, with AUC values of 0.71 and 0.7, respectively (Supplementary Fig. 6). Alterations in bacterial valine and leucine metabolism have previously been reported in the gut microbiome of SLE patiens [44]. However, the present study is the first to describe the alteration of this metabolism in the urobiome of these patients revealing differences between non-renal and renal SLE. Notably, the *ilvA* gene exhibited superior classification ability for renal SLE patients compared to the previously described 9-gene model. Its diagnostic application would also be advantageous, as it requires evaluating only a single biomarker.

The quantification analysis of relevant urine metabolites by NMR spectroscopy revealed a significant reduction in the urinary concentration of valine and leucine in non-renal SLE patients compared to controls (Fig. 7). However, this alteration was not observed in patients with renal SLE. While changes in these branched-chain amino acids (BCAAs) have been reported in other samples types, such as serum [45] or stool [44, 46], this is the first report documenting such alterations in urine. In addition, significant inverse correlations between *ilvH* or *ilvA* and the levels of some autoantibodies (anti-dsDNA, anti-U1-RNP, anti-SSA-60) suggest an involvement of altered microbiome-mediated amino acid degradation in the autoreactive immune response. These findings reveal that the pronounced urobiome dysbiosis observed in non-renal SLE patients may represent a compensatory mechanism for the inflammatory response, characterized by increased bacterial metabolic functions, including BCAA metabolism. Conversely, the urobiome of renal SLE individuals shows a significant loss of function, precluding such compensation.

## Conclusions

This study highlights the diagnostic potential of the urobiome in SLE, particularly through bacterial genes such as *ilvA* to differentiate between non-renal and renal SLE patients. While previous research has proposed intestinal taxonomic alterations for SLE or renal SLE [47, 48], the diagnostic power of bacterial genes, particularly in the urobiome, has not been explored. Urobiome-based biomarkers offer a non-invasive, rapid and cost-effective diagnostic approach.

These findings advance our understanding of the role of the urobiome in SLE, a multifaceted and complex disease, and open a new avenue for developing innovative, non-invasive diagnostic strategies.

## Supplementary Information

Supplementary Material 1. Supplementary Fig. 1. Variance explained by clinical variables. Bar plot showing the proportion of variance (R^2^) explained by potential cofounder factors analysed using PERMANOVA. Only variables that were significantly different between groups in univariate analysis (Mann–Whitney test or Fisher’s exact test) were included. R^2^ values are shown as percentages along with the corresponding *p*-values. Supplementary Fig. 2. Venn diagram of significant differentially enriched bacterial species identified by LEfSe analysis (*p* < 0.05, LDA > 2.0). Supplementary Fig. 3. Importances of the top-ranking features in the optimized taxa-based model. Supplementary Fig. 4. Receiver Operating Characteristic (ROC) curves for individual features in the valine and leucine biosynthesis pathway. Each curve represents the mean ROC across folds for a single feature. The Area Under the Curve (AUC) is shown in the legend. Supplementary Fig. 5. Comparison of AUC values obtained from tenfold cross-validation of a RF model trained using individual genes (ilvA and ilvH) and their combination. Significance was determined by one-way ANOVA (ns: not significant). Supplementary Fig. 6. Spearman correlations between gene-based biomarkers and clinical parameters. The heatmap represents Spearman’s rho values for pairwise correlations among SLEDAI, complement components (C3 and C4) and autoantibody levels. Asterisks indicate statistically significant correlations (*p* < 0.05). Supplementary Fig. 7. Differential alteration of the phenylalanine, tyrosine and tryptophan metabolism in non-renal and renal SLE patients. a) Phenylalanine, tyrosine and tryptophan biosynthesis pathway maps represented using KEGG Mapper Color tool. Differential gene abundance between non-renal or renal SLE and healthy controls was assessed by calculating logFC. Significant differences in gene abundances (*p*-adjusted < 0.05) between SLE groups and controls are coloured in the map with a color gradient and marked with stars. b) Mean AUC value of the RF model using differentially enriched genes of this pathway as biomarkers of renal SLE versus non-renal SLE. c) Receiver Operating Characteristic (ROC) curves for individual features in the phenylalanine, tyrosine and tryptophan biosynthesis pathway. Each curve represents the mean ROC across folds for a single feature. The Area Under the Curve (AUC) is shown in the legend.

Supplementary Material 2. Supplementary Table 1. Demographics and clinical characteristics of study participants in discovery and validation cohorts. Significance was determined by Mann–Whitney test, Kruskal–Wallis test or Fisher’s exact test. Supplementary Table 2. Primers used for gene validation by qPCR.

Supplementary Material 3. Supplementary Table 3. Statistically significant KOs between control and non-renal SLE group (padj < 0.05, LDA > 1.0). Supplementary Table 4. Statistically significant KOs between control and renal SLE group (padj < 0.05, LDA > 1.0). Supplementary Table 4. Statistically significant KOs between non-renal and renal SLE group (padj < 0.05, LDA > 1.0).

Supplementary Material 4. Code used to perform bioinformatics analyses.

## Data Availability

The raw sequencing data are available at the Sequence Read Archive (SRA) of the National Centre of Biotechnology Information (NCBI) under the Bioproject accession number PRJNA1237824.

## Abbreviations

SLE: Systemic lupus erythematosus
NMR: Nuclear magnetic resonance
RF: Random forest
PCR: Polymerase chain reaction
GFR: Glomerular filtration rate
rRNA: Ribosomal ribonucleic acid
DNA: Deoxyribonucleic acid
EDTA: Ethylenediaminetetraacetic acid
SRA: Sequence Read Archive
NCBI: National Center for Biotechnology Information
OTU: Operational taxonomic unit
PICRUSt: Phylogenetic Investigation of Communities by Reconstruction of Unobserved States
KEGG: Kyoto Encyclopedia of Genes and Genomes
LefSe: Linear discriminant analysis effect size
LDA: Linear discriminant analysis
KO: KEGG ortholog
FC: Fold change
AUC: Area under curve
