# Supplementary material for: Identification of urinary bacterial genes as biomarkers for non-invasive diagnosis of renal lupus
Source: Biomark Res. 2025 Sep 26;13:117. doi: 10.1186/s40364-025-00828-5 (PMC12465692; doi:10.1186/s40364-025-00828-5)
Supplement: Supplementary file 1 — Supplementary Material 1. Supplementary Fig. 1. Variance explained by clinical variables. Bar plot showing the proportion of variance (R2) explained by potential cofounder factors analysed using PERMANOVA. Only variables that were significantly different between groups in univariate analysis (Mann–Whitney test or Fisher’s exact test) were included. R2 values are shown as percentages along with the corresponding p-values. Supplementary Fig. 2. Venn diagram of significant differentially enriched bacterial species identified by LEfSe analysis (p < 0.05, LDA > 2.0). Supplementary Fig. 3. Importances of the top-ranking features in the optimized taxa-based model. Supplementary Fig. 4. Receiver Operating Characteristic (ROC) curves for individual features in the valine and leucine biosynthesis pathway. Each curve represents the mean ROC across folds for a single feature. The Area Under the Curve (AUC) is shown in the legend. Supplementary Fig. 5. Comparison of AUC values obtained from tenfold cross-validation of a RF model trained using individual genes (ilvA and ilvH) and their combination. Significance was determined by one-way ANOVA (ns: not significant). Supplementary Fig. 6. Spearman correlations between gene-based biomarkers and clinical parameters. The heatmap represents Spearman’s rho values for pairwise correlations among SLEDAI, complement components (C3 and C4) and autoantibody levels. Asterisks indicate statistically significant correlations (p < 0.05). Supplementary Fig. 7. Differential alteration of the phenylalanine, tyrosine and tryptophan metabolism in non-renal and renal SLE patients. a) Phenylalanine, tyrosine and tryptophan biosynthesis pathway maps represented using KEGG Mapper Color tool. Differential gene abundance between non-renal or renal SLE and healthy controls was assessed by calculating logFC. Significant differences in gene abundances (p-adjusted < 0.05) between SLE groups and controls are coloured in the map with a color gradient and marked [file 40364_2025_828_MOESM1_ESM.pdf]

Supplementary Figure 1

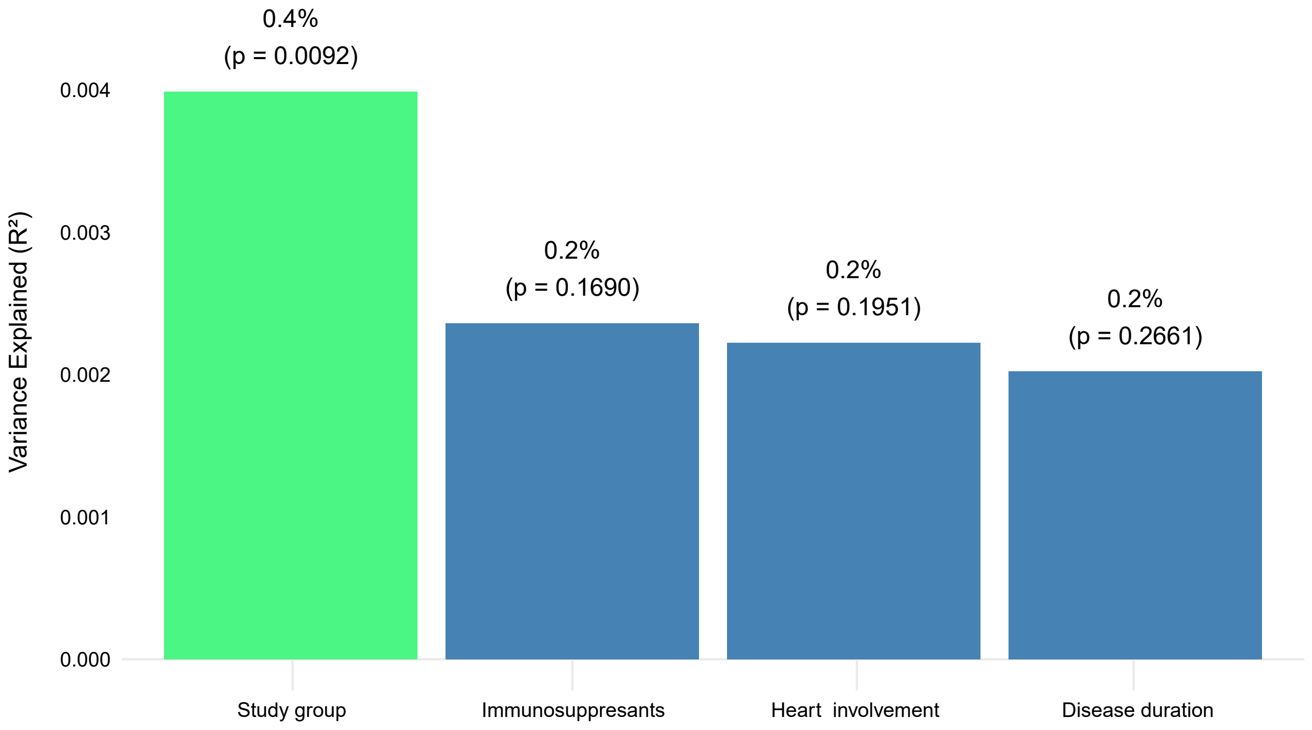

Supplementary Figure 2

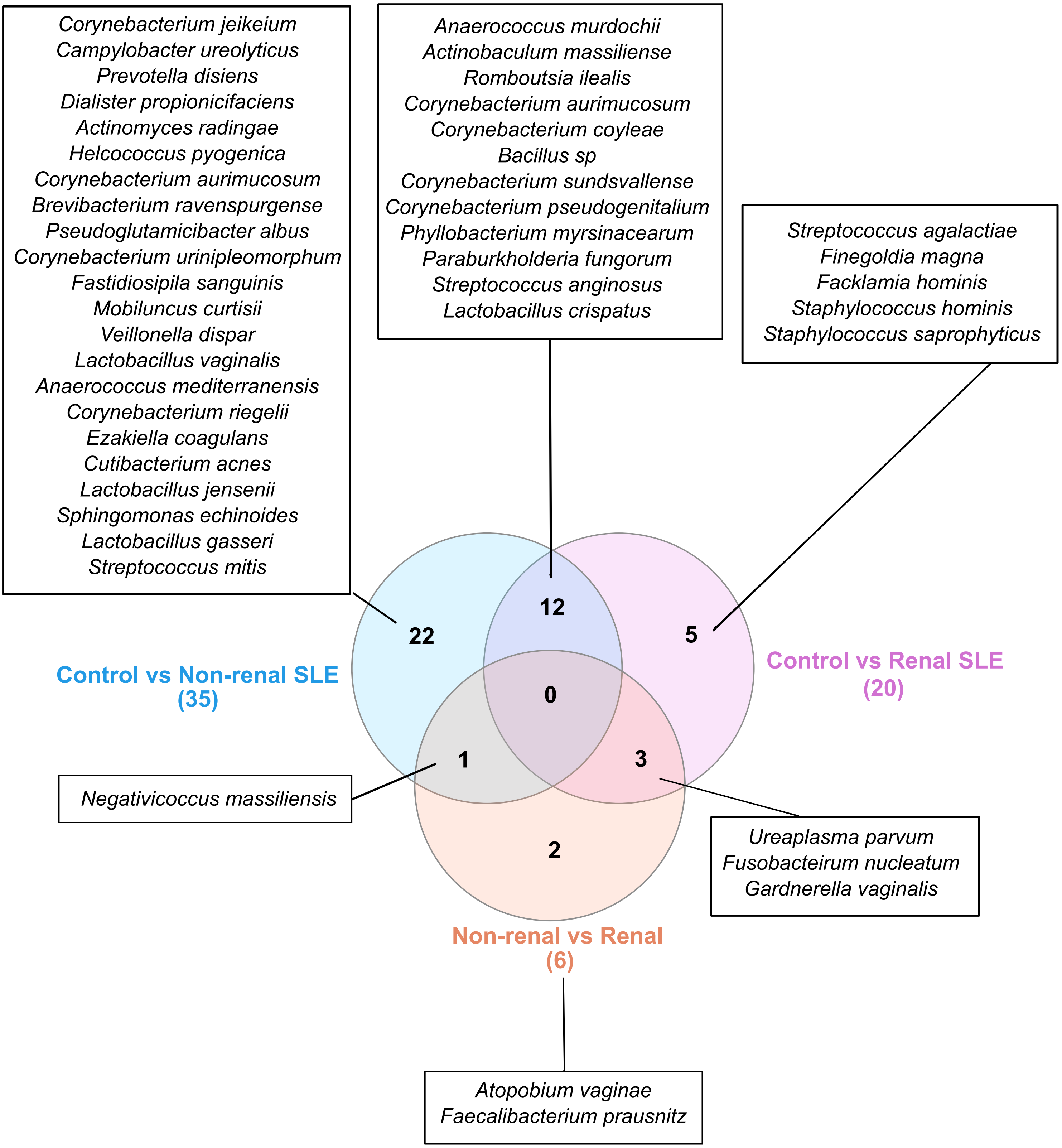

Supplementary Figure 3

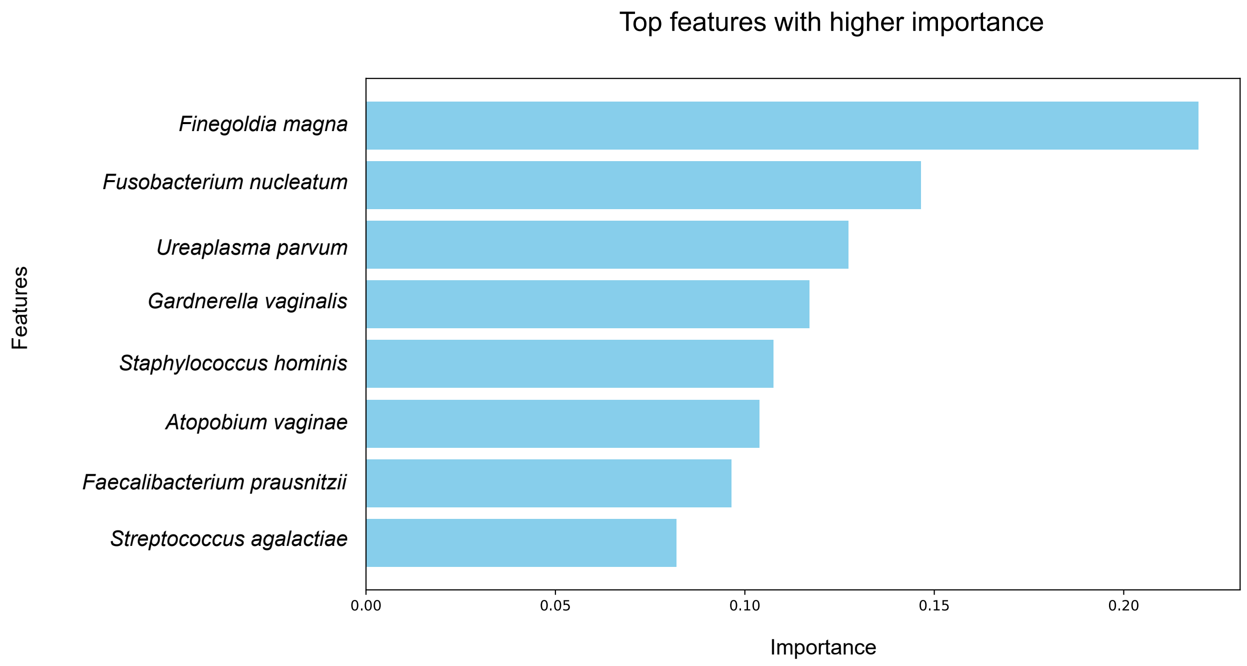

Supplementary Figure 4

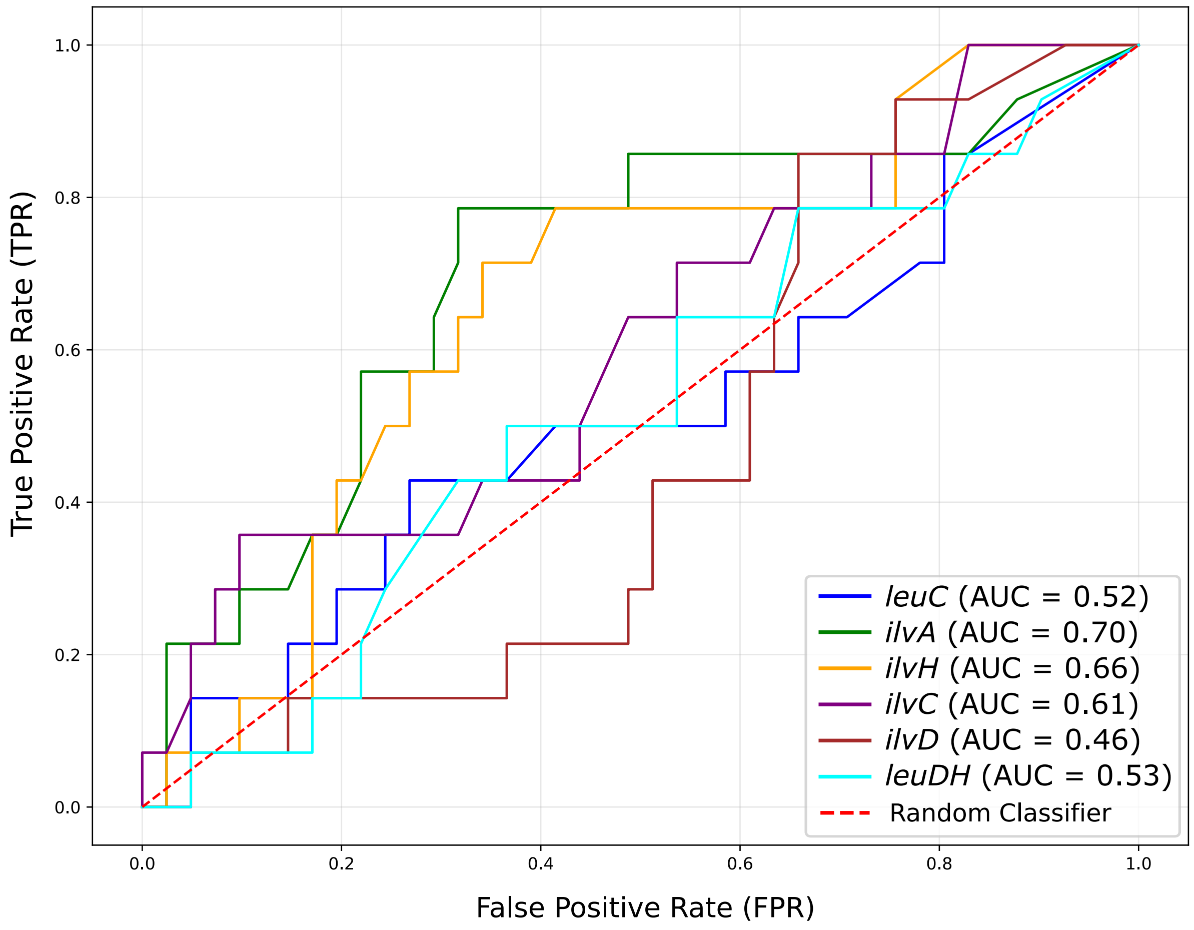

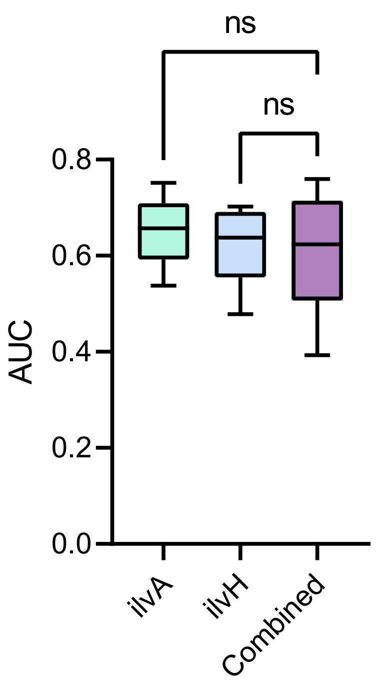
ns
ns
0.8
0.6
0.4
0.2
0.0
AUC
ilvA
ilvH
Combined

Supplementary Figure 6

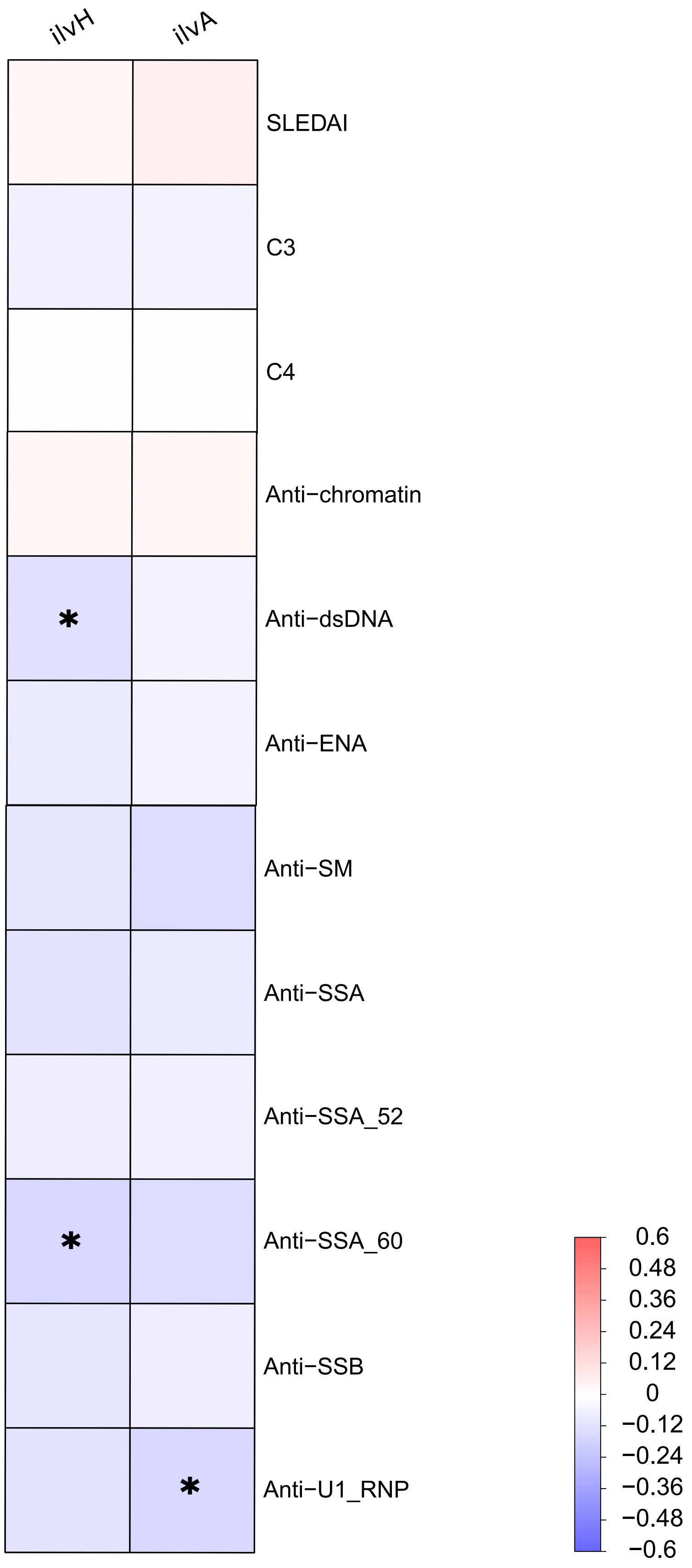

a

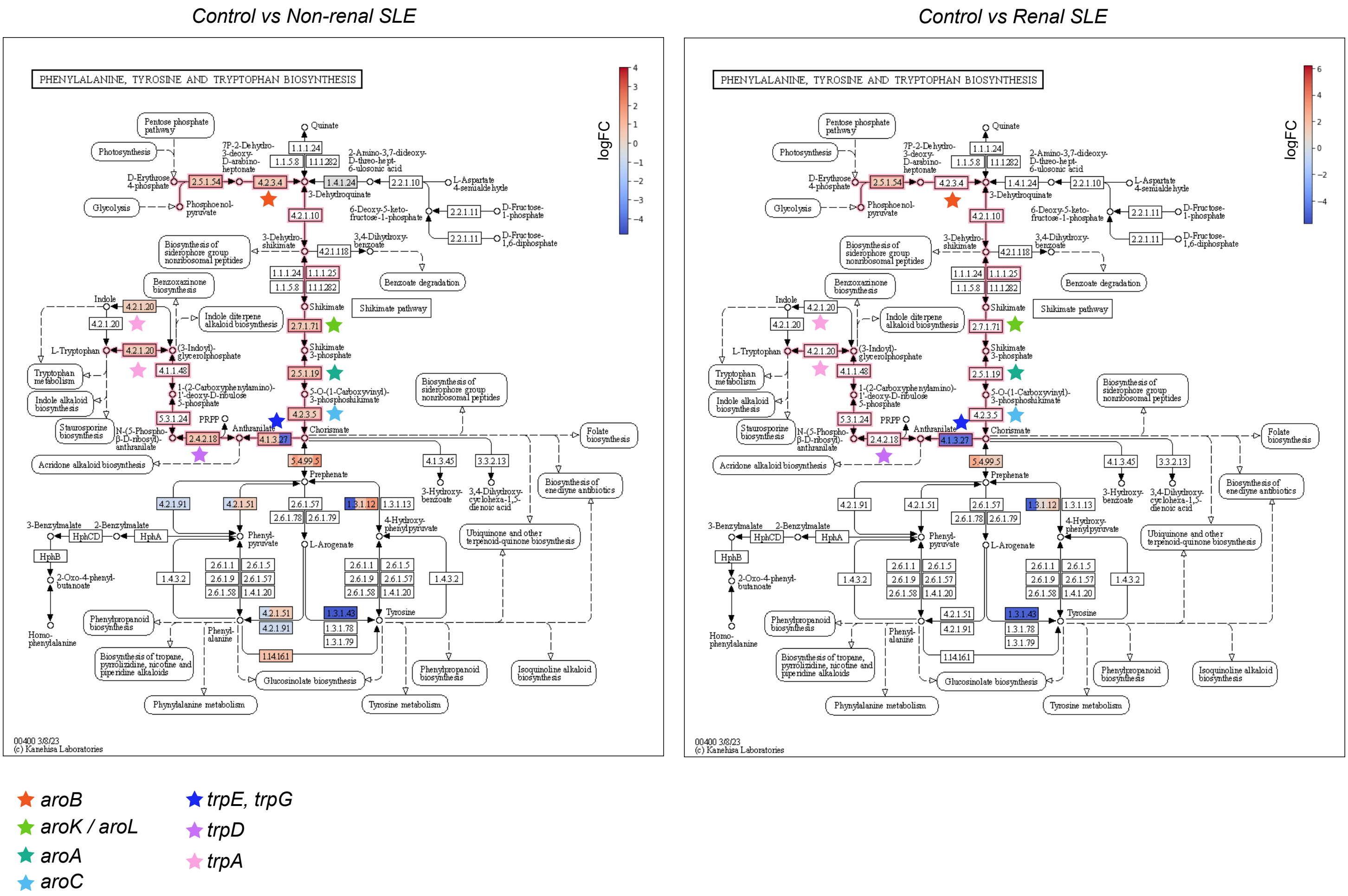
Control vs Non-renal SLE
Control vs Renal SLE
PHENYLALANINE, TYROSINE AND TRYPTOPHAN BIOSYNTHESIS
logFC
00400 3/8/23
(c) Kanehisa Laboratories
aroB
aroK / aroL
aroA
aroC
trpE, trpG
trpD
trpA

b

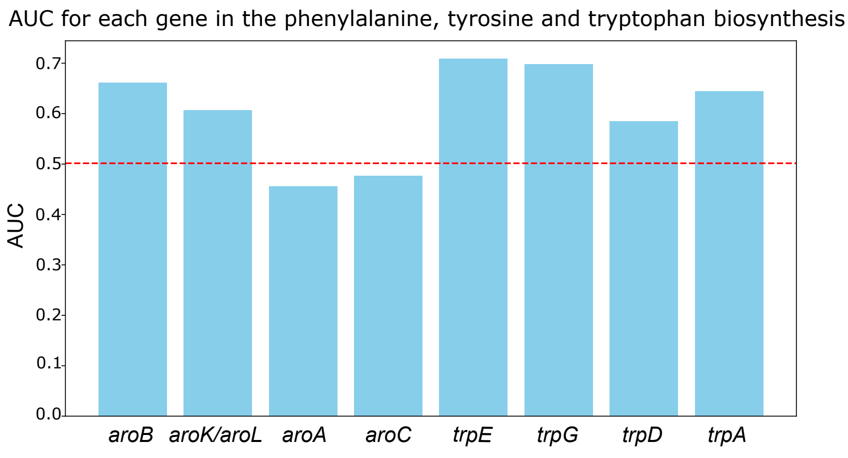
AUC for each gene in the phenylalanine, tyrosine and tryptophan biosynthesis
AUC
aroB
aroK/aroL
aroA
aroC
trpE
trpG
trpD
trpA

c

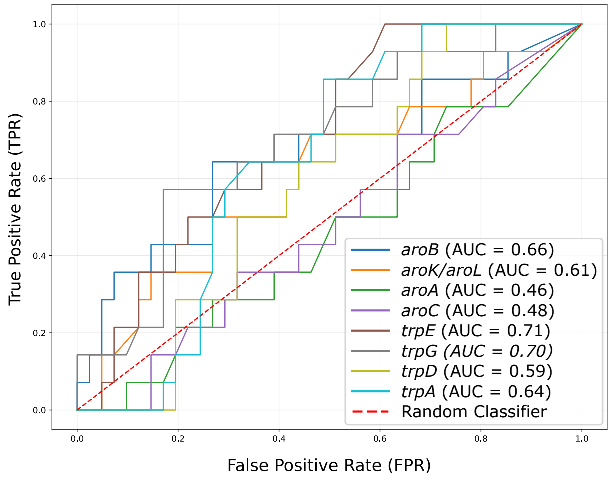
True Positive Rate (TPR)
False Positive Rate (FPR)
aroB (AUC = 0.66)
aroK/aroL (AUC = 0.61)
aroA (AUC = 0.46)
aroC (AUC = 0.48)
trpE (AUC = 0.71)
trpG (AUC = 0.70)
trpD (AUC = 0.59)
trpA (AUC = 0.64)
Random Classifier
