# Supplementary material for: Identification of urinary bacterial genes as biomarkers for non-invasive diagnosis of renal lupus
Source: Biomark Res. 2025 Sep 26;13:117. doi: 10.1186/s40364-025-00828-5 (PMC12465692; doi:10.1186/s40364-025-00828-5)
Supplement: Supplementary file 2 — Supplementary Material 2. Supplementary Table 1. Demographics and clinical characteristics of study participants in discovery and validation cohorts. Significance was determined by Mann–Whitney test, Kruskal–Wallis test or Fisher’s exact test. Supplementary Table 2. Primers used for gene validation by qPCR. [file 40364_2025_828_MOESM2_ESM.pdf]

Supplementary table 1.

	Healthy control	Non-renal SLE	Renal SLE	*P* value
Discovery cohort				
No. of patients	309	206	70	
Age (mean ± SD, years)	45.40 ± 12.6	45.58 ± 13.7	44.57 ± 12.68	0.5115
Female sex (n, %)	309 (100%)	206 (100%)	70 (100%)	
Ethnic origin (n, %)				*; 0.023
Caucasian/White	306 (99 %)	199 (97.1 %)	63 (90 %)	
Black/African American	0 (0%)	3 (1.5 %)	1 (1.4%)	
Asian	1 (0.3 %)	1 (0.5 %)	3 (4.3 %)	
Other	2 (0.6%)	2 (1 %)	3 (4.3 %)	
Obesity (n, %)	24 (7.8%)	10 (4.9%)	5 (7.1%)	0.5873
Smoking (n, %)	47 (15.2%)	41 (20%)	15 (21.4%)	0.4668
Disease duration (mean ± SD, years)	N/A	14.03 ± 10.26	17.7 ± 9.48	**; 0.002
Medications (n, %)				
Steroid usage	N/A	106 (51.7%)	45 (64.28%)	0.0746
Antimalarials	N/A	144 (70.24%)	44 (62.85 %)	0.2913
Immunosuppressants	N/A	57 (27.8%)	39 (55.7%)	***; 0.0001
Biologicals	N/A	0 (0%)	0 (0%)	>0.999
SLEDAI (mean ± SD, score)	N/A	5.02 ± 6.38	5.27 ± 6.18	0.8989
Organ involvement (n; %)				
Muscle-Skeletal	N/A	32 (15.5%)	9 (12.8%)	0.6794
Kidney	N/A	0 (0%)	70 (100%)	****; <0.0001
Skin and Mucosa	N/A	119 (57.77%)	41 (58.57%)	0.999
Heart	N/A	53 (25.73%)	37 (52.85%)	***; 0.0001
Lung	N/A	10 (4.85%)	5 (7.14%)	0.5370
Gastrointestinal	N/A	37 (17.9%)	16 (22.8%)	0.4751
Nervous system	N/A	7 (3.4 %)	4 (5.7 %)	0.4869
Vascular	N/A	94 (45.6%)	41 (58.57%)	0.0876
Serological profile				
Low C3 (n, %)	N/A	66 (32.04%)	20 (28.57%)	0.6429
Low C4 (n, %)	N/A	43 (20.87%)	14 (20%)	0.999
Anti-dsDNA (mean ± SD)*	1.5 ± 4.75	49.04 ± 98.16	117.4 ± 188.9	****; <0.0001
Anti-Chromatin (mean ± SD)*	16.2 ± 34.7	215.6 ± 247.3	267.8 ± 271.5	****; <0.0001
Anti-ENA (mean ± SD)*	0.26 ± 0.42	4.33 ± 5.94	2.51 ± 3.92	****; <0.0001
Anti-SM (mean ± SD)*	0 ± 0	6.14 ± 37.9	4.32 ± 15.9	****; <0.0001
Anti-SSA (mean ± SD)*	2.83 ± 40.5	156 ± 249.3	95.5 ± 201.9	****; <0.0001
Anti-SSA_52 (mean ± SD)*	0.26 ± 4.2	119.3 ± 229.6	63.5 ± 167.7	****; <0.0001
Anti-SSA_60 (mean ± SD)*	2.56 ± 40.4	140.3 ± 247.5	105.5 ± 216	****; <0.0001
Anti-SSB (mean ± SD)*	0.002 ± 0.03	30.7 ± 125.3	3.24 ± 17.8	****; <0.0001
Anti-U1_RNP (mean ± SD)*	0.22 ± 1.68	13.67 ± 41.68	3.39 ± 7.64	****; <0.0001
Validation cohort				
No. of patients	30	30	30	
Age (mean ± SD, years)	48.69 ± 10.8	42.58 ± 14.7	41.96 ± 15.3	0.06
Female sex (n, %)	30 (100%)	30 (100%)	30 (100%)	
Ethnic origin (n, %)				*; 0.0156

Caucasian/White	29 (96.7%)	29 (96.7%)	27 (90%)	
Black/African American	0 (0%)	0 (0%)	2 (6.7%)	
Asian	0 (0%)	0 (0%)	0 (0%)	
Other	1 (3.3%)	1 (3.3%)	1 (3.3%)	
Obesity (n, %)	3 (10%)	4 (13.3%)	2 (6.7%)	0.2436
Smoking (n, %)	5 (16.7%)	6 (20%)	3 (10%)	0.1416
Disease duration (mean ± SD, years)	NA	11.8 ± 13.7	30.3 ± 30.4	**; 0.0024
Medications (n, %) †				
Steroid usage	NA	11 (36.7%)	9 (30%)	0.3404
Antimalarials	NA	24 (80%)	11 (67.7%)	0.1412
Immunosuppressants	NA	7 (23.3%)	10 (58%)	****; <0.0001
Biologicals	NA	0 (0%)	0 (0%)	>0.999
SLEDAI (mean ± SD, score)	NA	17.8 ± 12.8	21.1 ± 16.6	0.7824
Organ involvement (n; %)				
Muscle-Skeletal	NA	7 (23.3%)	4 (13.36%)	0.0963
Kidney	NA	0 (0%)	30 (100%)	****; <0.0001
Skin and Mucosa	NA	20 (66.7%)	16 (53.3%)	0.0813
Heart	NA	4 (13.3%)	13 (43.3%)	****; <0.0001
Lung	NA	3 (10%)	2 (6.67%)	0.4353
Gastrointestinal	NA	6 (20%)	6 (20%)	>0.999
Nervous system	NA	1 (3.3%)	0 (0%)	0.2462
Vascular	NA	15 (50%)	14 (46.7%)	0.6712
Serological profile				
Anti-dsDNA (mean ± SD)	NA	69.87 ± 157.8	73 ± 92.3	0.6325
Low C3 (n, %)	NA	4 (13.3%)	4 (13.3%)	>0.999
Low C4 (n, %)	NA	3 (10%)	2 (6.7%)	0.4353

* No significant (ns) differences between non-renal and renal SLE patients (Kruskal-Wallis test)

† Data were missing from 10 renal SLE patients.

Supplementary Table 2.

Gene name	KO number	Primer forward (sense)	Primer reverse (antisense)
IlvC	K00053	AAT TGT ACG AAG CAG AAA TCG C	GGT CCT TTA GGA GCA CAC AT
leuC	K01703	TGA TGA GCG AGC CTA CAA TTA	TGC AAG TCA CTG AGA CGA G
ilvA	K01754	GGG ATT GAT TTC TGA AAC CTT G	TCC TCC AGA AAT GAT ACA ACA A
ilvH	K01653	TAC GGA TAA ACC ACA CTT GGA G	GTT GCA CGG AAA GGT TGA ATA A
ilvD	K01687	TCG TTG ATG GCG ATG TAG TC	ACC TTG TCT CCT TGA CCT TTA C
LeuDH	K00263	CTC GCC ACC AAG GAA GAT G	TTC TAG GCC GAC ATT GAT GAT G
pimC	K14335	ACT CGG TCG TGG CTA TCT	CCG GAT CTC GGC AAC TAT C
sco1	K07152	CAA TAT GTC CCG GCC TTC C	CCA TCG GCC TTG CGA TAA TA
selA	K01042	GAT GCC CTG GTC GTC AAT AA	AAA GAC CCG CCG ATT TCA
ABC.CD.A	K02003	GCC TAA TCT AAC AGT CAG ACA GAA	CCA AGT CCC AAA TCC TCG ATA A
ABCB-BAC	K06147	CCT CAT CAG CAG TTA TTT GAA GG	CTG TAT TAA CTG ACC CAC TCC A
transposase	K07483	TAT CAT GAA CAT CGG GAA CTC G	GCA TTT CGC CAT TGT CCT TC
ABC.CD.P	K02004	AGT CGA GAC TTG GTG ATT GG	GTC ATA GAA ACA CGA GAT TCT TTC TG
cysK	K01738	TGG TAG CGA AGG AAT GAA AGG	GTG TAC TTC TGG ATT AGC TGG ATT
lldG	K00782	AAG AAA TCG GCG CTG TAG AC	GCC ATA ATC GGC TTC GGT AA
16S		AAA CTC AAA KGA ATT GAC GGG G	GGG TTG CGC TCG TTR YGG
